# Dextrose solution for priming and rinsing the extracorporeal circuit in hemodialysis patients: A prospective pilot study

**DOI:** 10.1177/03913988211020023

**Published:** 2021-05-31

**Authors:** Paul A Rootjes, Erik Lars Penne, Georges Ouellet, Yanna Dou, Stephan Thijssen, Peter Kotanko, Jochen G Raimann

**Affiliations:** 1 Department of Nephrology, Northwest Clinics, Alkmaar, The Netherlands; 2 Department of Nephrology, Amsterdam University Medical Centers, location AMC, Amsterdam, The Netherlands; 3 Hôpital Maisonneuve-Rosemont, Montréal, QC, Canada; 4 The Nephrology Center, The First Affiliated Hospital of Zhengzhou University, Henan, China; 5 Renal Research Institute, New York, NY, USA; 6 Icahn School of Medicine at Mount Sinai, New York, NY, USA

**Keywords:** Hemodialysis, artificial kidney, apheresis and detoxification techniques, dialysis fluids, isotonic saline, dextrose 5%, sodium loading, interdialytic weight gain, extracorporeal circuit, priming and rinsing, thirst

## Abstract

**Introduction:**

Excess sodium intake and consequent volume overload are major clinical problems in hemodialysis (HD) contributing to adverse outcomes. Saline used for priming and rinsing of the extracorporeal circuit is a potentially underappreciated source of intradialytic sodium gain. We aimed to examine the feasibility and clinical effects of replacing saline as the priming and rinsing fluid by a 5% dextrose solution.

**Materials and methods:**

We enrolled non-diabetic and anuric stable HD patients. First, the extracorporeal circuit was primed and rinsed with approximately 200–250 mL of isotonic saline during 4 weeks (Phase 1), subsequently a similar volume of a 5% dextrose solution replaced the saline for another 4 weeks (Phase 2), followed by another 4 weeks of saline (Phase 3). We collected data on interdialytic weight gain (IDWG), pre- and post-dialysis blood pressure, intradialytic symptoms, and thirst.

**Results:**

Seventeen chronic HD patients (11 males, age 54.1 ± 18.7 years) completed the study. The average priming and rinsing volumes were 236.7 ± 77.5 and 245.0 ± 91.8 mL respectively. The mean IDWG did not significantly change (2.52 ± 0.88 kg in Phase 1; 2.28 ± 0.70 kg in Phase 2; and 2.51 ± 1.2 kg in Phase 3). No differences in blood pressures, intradialytic symptoms or thirst were observed.

**Conclusions:**

Replacing saline by 5% dextrose for priming and rinsing is feasible in stable HD patients and may reduce intradialytic sodium loading. A non-significant trend toward a lower IDWG was observed when 5% dextrose was used. Prospective studies with a larger sample size and longer follow-up are needed to gain further insight into the possible effects of using alternate priming and rinsing solutions lowering intradialytic sodium loading.

**Trial registration:**

Identifier NCT01168947 (ClinicalTrials.gov).

## Introduction

Excess sodium and subsequent volume overload are major clinical problems in hemodialysis (HD) patients and have been associated with adverse outcomes. On a short term, this may lead to high ultrafiltration (UF) rates and subsequent intradialytic complications and hypertension,^1,2^ while on a long term, this may result in left ventricular hypertrophy, congestive heart failure,^3^ and increased mortality.^4–6^

Several sources of excess sodium or sodium loading have been identified in these patients.^7^ The major source of sodium loading comes from the frequent nonadherence to the recommended sodium restricted diet. The dietary sodium intake can be quite excessive and reportedly amount of up to 10 g of salt.^8^ Another substantial source of sodium comes from the dialysis treatment itself, for example in the presence of a positive dialysate-to-serum sodium gradient leading to intradialytic diffusive sodium loading, or if saline solutions with sodium concentrations greater than the plasma sodium (normally saline solutions have sodium concentrations at around 154 mEq/L) are administrated during HD. The former may occur if the dialysate sodium concentration is higher than the serum sodium (dialyzing against a positive sodium gradient), and when certain sodium profiles are used for the prevention of intradialytic hemodynamic instability. The latter may occur when saline boluses are administrated to prevent or treat intradialytic symptoms.^7–12^

Analogously, the solution used for priming and rinsing of the dialyzer and blood lines is an underappreciated source of intradialytic sodium loading.^13^ At the beginning and at the end of every standard HD session, the dialyzer and blood lines are generally primed and rinsed with 200–250 mL of isotonic saline solution (0.9%). Thus, a volume up to 500 mL of saline (containing 77 mmol of sodium) is infused into the patient each treatment. Priming and rinsing practices could theoretically contribute to sodium loading, also when the infused volume used is accounted for by additional ultrafiltration. As a resultant of the additional sodium loading, the perceived thirst increases and patients will drink more fluids following the dialysis treatment and therefore increase the risk of intradialytic symptoms due to the need for additional excess fluid removal.^13^

We hypothesized that the replacement of isotonic saline as the priming and rinsing solution by an identical volume of a 5% dextrose solution will result in a reduction of sodium loading. This pilot study investigated the feasibility of this approach in a US clinic and analyzed the effects on interdialytic weight gain (IDWG), blood pressure, and self-reported thirst.

## Materials and methods

### Study setting

We conducted this study as a non-randomized, cross-over, interventional study (clinicaltrials.gov identifier NCT01168947), investigating the effect of removing sodium loading from the process of priming and rinsing. While designed as a pilot study, we also investigated the effects on IDWG as the primary outcome, and pre- and post-dialysis blood pressure (BP), intradialytic events, and self-reported thirst as the secondary outcomes. Patients were recruited at the Avantus Renal Therapies clinic in New York, NY, USA; and considered eligible if they were older than 18 years of age, ambulatory, had no diabetes, and considered clinically stable on a thrice weekly HD regimen. Predefined exclusion criteria included residual kidney function (urine volume greater than 500 mL per day), a life expectancy less than 6 months, any psychological condition that could interfere with the patient’s ability to comply with the study protocol, the expectation that the native kidney function would recover and scheduled for living donor kidney transplant.

The study was conducted compliant with the Declaration of Helsinki, approved by the Medical Ethical Committee of the Beth Israel Medical Center, NY, USA. All participants gave informed consent prior to entering the study.

### Study design

**Figure 1.**
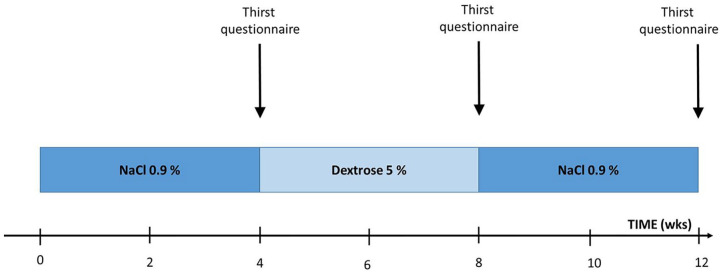
Study flowchart. The solution for priming and rinsing was changed during the study. In weeks 1–4 (Phase 1) saline 0.9% was used; in weeks 5–8 (Phase 2) a dextrose 5% solution; and in weeks 9–12 (Phase 3) the priming and rinsing solution was switched back to saline 0.9%. At the time points blood pressure was measured under controlled conditions and thirst questionnaires were completed. wks: weeks; NaCl 0.9%: sodium chloride 0.9%.

The study consisted of three phases, 4 weeks each (Figure 1). In Phase 1 and Phase 3, hemodialysis was performed as per standard of care. During these periods the extracorporeal circuit (dialyzer and blood lines) was primed with approximately 200–250 mL of isotonic saline (0.9% with a 154 mEq/L sodium concentration) before connection to the patient’s dialysis access. At the end of the treatment, the dialysis circuit was rinsed with 200–250 mL of isotonic saline. In accordance to the clinics’ routine care, the priming and rinsing volume, a combined volume at around 500 mL, was infused into the patients during treatment. Consequently, the ultrafiltration goal was adjusted to include this excess fluid. In Phase 2, the solution for priming and rinsing was replaced by similar volumes of a dextrose 5% solution. No other modifications to the dialysis treatment, including dry weight and the dialysate composition or to the patient’s prescribed medications were allowed.

### Measurements

During the 12-weeks study period, we obtained pre- and post-dialysis weight, systolic BP (SBP), and diastolic BP (DBP) from each HD session.^14^ The absolute interdialytic weight gain (IDWG) in kilograms (kg) and the relative IDWG expressed as percentage (%) of body weight (IDWG/post-HD weight) were calculated. Further, we recorded intradialytic symptoms, including hypotensive episodes. For the evaluation of perceived thirst, we administrated a standardized thirst questionnaire (Dialysis Thirst Inventory^15^) at the end of each phase before the first dialysis of the next phase. The questionnaire consists of seven items self-reporting thirst on a five-point Likert scale (never = 1, to very often = 5). The sum of scores provides an overall thirst score ranging from 7 (no thirst) to 35 (very thirsty). The questions were posed as follows: (1) Thirst is a problem for me; (2) I am thirsty during the day; (3) I am thirsty during the night. (4) My social life is influenced. (5) I am thirsty before dialysis. (6) I am thirsty during dialysis. (7) I am thirsty after dialysis. Essential baseline patient data were retrieved from medical files.

### Statistical analyses

Due to the nature of this study as a pilot project, we had not conducted a formal power calculation but decided for a recruitment target of 20 patients. Data are presented as means with standard deviations for normally distributed variables or median with interquartile ranges (IQR) for non-normally distributed variables. Differences between groups were tested using one-way within-groups ANOVA or Chi-square tests, where appropriate. A *p*-value less than 0.05 was considered statistically significant. Statistical analyses were conducted with IBM Statistical Package for Social Sciences (SPSS^®^) version 20.0 (IBM USA, Armonk, NY, USA).

## Results

### Study population

We studied 17 HD patients (11 males and 6 females, age 54.1 ± 18.7 years, 11 blacks, 3 whites and 3 Hispanics) with no residual kidney function. The mean treatment time of each hemodialysis session was 204 ± 23 min during the entire study; 203 ± 23, 205 ± 23 and 206 ± 25 min during phase 1, 2, and 3 respectively. We administered an average priming volume of 236.7 ± 77.5 mL and rinsing volume of 245.0 ± 91.8 mL (equal for saline and dextrose 5%).

### Interdialytic weight gain

**Figure 2.**
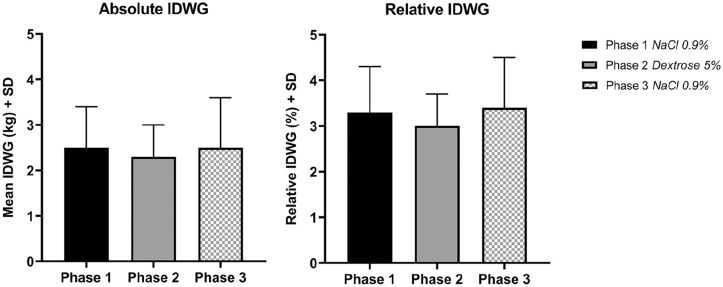
Absolute and relative IDWG in the three Phases. The mean absolute interdialytic weight gain (IDWG) in kilograms and the relative IDWG change as percentage (%) of body weight (IDWG/post-dialysis weight) among the 3 study phases are expressed with standard deviation (SD). Phase 1: NaCl 0.9%; Phase 2: Dextrose 5%; Phase 3: NaCl 0.9%.

As shown in Table 1 and Figure 2, the mean absolute IDWG during Phase 2 was slightly lower compared to Phase 1 and 3 (2.28 ± 0.70 vs. 2.52 ± 0.88 and 2.51 ± 1.12 kg respectively). The pre-dialysis weight in phase 2 was non-significantly lower compared to Phase 1 and 3 (79.3 ± 19.5 vs 79.8 ± 19.7 and 79.8 ± 19.6 kg respectively). The post dialysis weight was almost the same during Phase 1, 2, and 3 (77.3 ± 19.1, 77.0 ± 19.1, and 77.2 ± 19.0 kg). Although not statistically significant, the absolute and relative IDWG (Table 1) showed a non-significant decrease during Phase 2 as compared to Phase 1 and 3.

**Table 1.** Weight, blood pressure and DTI during the saline and dextrose phases.

	Phase 1	Phase 2	Phase 3	*p*-Value
Pre-HD Wt (kg)	79.8 ± 19.7	79.3 ± 19.5	79.8 ± 19.6	n.s.
Post-HD Wt (kg)	77.3 ± 19.1	77.0 ± 19.1	77.2 ± 19.0	n.s.
Absolute IDWG (kg)	2.52 ± 0.88	2.28 ± 0.70	2.51 ± 1.12	n.s.
Relative IDWG (%)	3.31 ± 1.04	3.02 ± 0.73	3.36 ± 1.10	n.s.
Pre-HD SBP (mmHg)	144.5 ± 16.9	142.5 ± 15.3	144.4 ± 14.3	n.s.
Pre-HD DBP (mmHg)	77.0 ± 11.8	77.8 ± 11.2	78.6 ± 15.6	n.s.
Post-HD SBP (mmHg)	132.7 ± 19.5	135.2 ± 17.6	139.6 ± 16.0	n.s.
Post-HD DBP (mmHg)	72.0 ± 13.8	73.6 ± 12.3	75.3 ± 13.1	n.s.
DTI-score	17.1 ± 5.4	16.9 ± 5.2	17.0 ± 4.0	n.s.

n.s.: not statistically significant.

The mean ± standard deviation (SD) of the pre- and post-dialysis weight (pre, post-HD Wt); the absolute IDWG (interdialytic weight gain) in kilograms (kg) and the relative IDWG expressed as a percentage (%) of bodyweight (IDWG/post-HD Wt); and the pre- and post-HD systolic and diastolic blood pressure (pre, post-HD SBP and DBP) are shown. Phase 1: NaCl 0.9%; Phase 2: Dextrose 5%; Phase 3: NaCl 0.9%.

The mean dialysis thirst inventory (DTI) score consisted of a five-point Likert scale (1 = never thirsty, 2 = almost never thirsty, 3 = occasional thirsty, 4 = fairly often thirsty, 5 = very often thirsty means).

### Pre- and post-dialysis blood pressures

As shown in Table 1, mean systolic (SBP) and diastolic blood pressure (DBP) before hemodialysis were not significantly different during Phase 1, 2, and 3 (144.5 ± 16.9, 142.5 ± 15.3, 144.4 ± 14.3, and 77.0 ± 11.8, 77.8 ± 11.2, 78.6 ± 15.6 mmHg respectively). Both the SBP and DBP after dialysis were slightly higher during Phase 3 compared to Phase 1 and 2 (139.6 ± 16.0 and 75.3 ± 13.1 vs 132.7 ± 19.5, 72.0 ± 13.8; 135.2 ± 17.6, 73.6 ± 12.3 mmHg respectively) without reaching statistical significance.

### Level of thirst

As depicted in Table 1, the total Dialysis Thirst Inventory (DTI) score did not differ between the three phases (Phase 1: 17.1 ± 5.4 vs. 16.9 ± 5.2 vs. 17.0 ± 4.0 during Phase 2 and 3 respectively). Therefore, an ancillary analysis was performed to compare the thirst scores more specific after and before hemodialysis among the different phases (data not shown). This analysis also showed no differences in the thirst scores before and after hemodialysis among the different phases.

### Intradialytic events

During the entire study period, only one patient experienced intradialytic symptoms. This hypotensive episode occurred once during phase 1 and was managed with a bolus of 300 mL of saline. No other adverse events were reported.

## Discussion

We assessed the relationship between the sodium content of the priming and rinsing solution and IDWG, blood pressure, and thirst in anuric, non-diabetic stable HD patients in this pilot study.

Replacing saline by a dextrose 5% solution for priming and rinsing appears to be feasible and resulted in a non-significant 0.2 kg IDWG reduction. Notably, this difference in IDWG was similar to the volume that was used for rinsing.

A recent randomized controlled trial investigated the effect of low sodium dialysate (135 mmol/l) versus conventional dialysate (140 mmol/l).^16^ In this study it was demonstrated that a negative sodium balance during hemodialysis, by lowering the sodium concentration, resulted in decreased interdialytic weight gain and extracellular volume. While low sodium dialysate did not reduce left ventricular mass after 12 months, a positive effect of intradialytic sodium loading is suggested based on these data. In addition, a clinical trial in 15 thrice-weekly-in-center nocturnal hemodialysis patients in whom the dialysate sodium was reduced resulted in a significant decrease in IDWG, post-dialysis plasma sodium concentration and pre-dialysis SBP.^17^

To the best of our knowledge, there is only one previous study that addressed the use of dextrose instead of saline for priming and rinsing of the extracorporeal circuit, with a focus on reducing intradialytic symptoms.^13^ In this study, 38 patients were switched from standard saline to a dextrose 5% solution for priming and rinsing of the extracorporeal circuit and also, instead of saline, for boluses to treat intradialytic symptoms. The authors report that IDWG decreased significantly by 0.2 kg 1 week after the switch from saline to dextrose. However, this effect was no longer observed after 1 month. This study was not randomized or controlled, and did also not have any form of cross-over design, therefore all results have to be interpreted with caution.^13^

By lowering the sodium content of the priming and rinsing fluid the net sodium removal during dialysis treatment can be increased. For instance, when a total volume of 400 mL saline 0.9% (containing 154 mmol/L of sodium) is used for priming and rinsing of the system is replaced by a similar volume of dextrose 5% (contains no sodium), then up to 61.6 mmol (0.4 × 154) or 1.4 g of sodium can additionally be removed per treatment at equal ultrafiltration volume. Notably, this 1.4 g of sodium represent more than 50% of the daily recommended dietary intake. However, due to diffusive sodium transfer between the dialysate and the blood during dialysis, the sodium lowering effect of dextrose 5% will be most pronounced during rinsing. Further, it also needs to be taken into account that the excess of sodium which is administrated during rinsing can only be removed during the following dialysis treatment. When designing the study, we hypothesized that the replacement of both priming and rinsing saline solution by a dextrose solution, would result in lower thirst, lower IDWG, and lower blood pressure. While this hypothesis was not confirmed in the current study, we believe the observation that the absolute IDWG was 0.2 kg lower during Phase 2 suggests a need for further studies.

In the present study, the rinsing procedure as described in the methods section was conducted according to the clinics’ routine care. In many other centers around the world blood can be returned to the patient by disconnecting with “air,” thereby preventing fluid administration. The latter procedure is prohibited in our clinic and in many other US clinics.

State-of-the-art dialysis machines are capable of producing on-line prepared solutions with a much lower sodium concentration than saline for priming and rinsing of the dialyzer and bloodlines.^18^ Hence, there is no need for additional saline solution bags anymore. The required amount of the priming and rinsing fluid is determined more precisely by the machine, thereby the volume is reduced to what is actually required. Notably, the sodium content of these on-line prepared solutions is lower than that of isotonic saline.^19^ Although this seems to be a positive development in reducing sodium loading, most machines still use a considerable fluid volume for priming and rinsing. Modern dialysis machines can adjust the dialytic sodium balance using electrolyte balancing modules.^20,21^ Future machines are expected to have the capability for an even more sophisticated adjustment of dialysate composition that includes both electrolytes and non-electrolytes.^21^

Our pilot study was limited by the small sample size and short follow-up. As a consequence, no conclusions regarding the perceived thirst and changes in BP related to the solution used for priming and rinsing can be drawn. Further, we only included stable and non-diabetic HD patients, thus the interpretability of the data on intradialytic symptoms is limited. In addition, a dextrose 5% solution contains calories, the caloric impact (170 kcal/L) is however limited. Furthermore, we could not discriminate separate effect of priming and rinsing. Also, we did not investigate whether a reduction in IDWG, perceived thirst and BP (reflecting a reduction in sodium loading) could also be achieved by just lowering the dialysate sodium concentration rather than priming the extracorporeal system with D5 instead of saline. Moreover, we did not study the possible equilibration between the priming fluid and the dialysate. Finally, the effects on hemodynamic stability definitely need to be addressed in future studies.

In conclusion, in our pilot study it appeared to be feasible to replace saline by a dextrose 5% solution for priming and rinsing. Moreover, this study suggests that IDWG may be reduced by lowering the sodium content of the fluid used for priming and rinsing. Additional studies with larger sample sizes are needed to confirm and extend on the interpretation of these pilot data. We believe that using dextrose solution for priming and rinsing instead of saline could be a valuable approach, especially in patients who have difficulties in achieving their optimal post-dialysis weight with minimal fluid overload. Although absolute differences in IDWG by changing priming and rinsing solution may be small, this might have substantial impact on a larger scale.

## References

[1] Raimann J Liu L Tyagi S , et al. A fresh look at dry weight. Hemodial Int 2008; 12: 395–405. 19090861 10.1111/j.1542-4758.2008.00302.x

[2] Thijssen S Raimann JG Usvyat LA , et al. The evils of intradialytic sodium loading. Contrib Nephrol 2011; 171: 84–91. 21625095 10.1159/000327333

[3] Fagugli RM Pasini P Quintaliani G , et al. Association between extracellular water, left ventricular mass and hypertension in haemodialysis patients. Nephrol Dial Transplant 2003; 18: 2332–2338. 14551362 10.1093/ndt/gfg371

[4] Kalantar-Zadeh K Regidor DL Kovesdy CP , et al. Fluid retention is associated with cardiovascular mortality in patients undergoing long-term hemodialysis. Circulation 2009; 119: 671–679. 19171851 10.1161/CIRCULATIONAHA.108.807362 PMC2773290

[5] Pinter J Chazot C Stuard S , et al. Sodium, volume and pressure control in haemodialysis patients for improved cardiovascular outcomes. Nephrol Dial Transplant 2020; 35(Suppl. 2): ii23–ii30. 10.1093/ndt/gfaa017 PMC7066545 32162668

[6] Hecking M Moissl U Genser B , et al. Greater fluid overload and lower interdialytic weight gain are independently associated with mortality in a large international hemodialysis population. Nephrol Dial Transplant 2018; 33(10): 1832–1842. 29688512 10.1093/ndt/gfy083 PMC6168737

[7] Penne EL Levin NW Kotanko P. Improving volume status by comprehensive dietary and dialytic sodium management in chronic hemodialysis patients. Blood Purif 2010; 30: 71–78. 20616547 10.1159/000317124

[8] Mc Causland FH Waikar SS Brunelli SM. The relevance of dietary sodium in hemodialysis. Nephrol Dial Transplant 2013; 28(4): 797–802. 23129821 10.1093/ndt/gfs452 PMC3716331

[9] Mann H Stiller S. Sodium modeling. Kidney Int Suppl 2000; 58(76): S79–S88. 10936803

[10] Lee SW. Sodium balance in maintenance hemodialysis. Electrolyte Blood Press 2012; 10(1): 1–6. 23508564 10.5049/EBP.2012.10.1.1 PMC3597912

[11] Hanafusa N Tsuchiya K Nitta K. Dialysate sodium concentration: the forgotten salt shaker. Semin Dial 2018; 31(6): 563–568. 30343516 10.1111/sdi.12749

[12] Hecking M Karaboyas A Saran R , et al. Predialysis serum sodium level, dialysate sodium, and mortality in maintenance hemodialysis patients: the Dialysis Outcomes and Practice Patterns Study (DOPPS). Am J Kidney Dis 2012; 59(2): 238–248. 21944663 10.1053/j.ajkd.2011.07.013

[13] Rayment G San Miguel S Chow J. Sugar or salt: the use of 5% dextrose in the adult, non-diabetic haemodialysis population. Renal Soc Aust J 2013; 9(2): 58–66.

[14] Perloff D Grim C Flack J , et al. Human blood pressure determination by sphygmomanometry. Circulation 1993; 88: 2460–2470. 8222141 10.1161/01.cir.88.5.2460

[15] Bots CP Brand HS Veerman ECI , et al. Interdialytic weight gain in patients on hemodialysis is associated with dry mouth and thirst. Kidney Int 2004; 66: 1662–1668. 15458464 10.1111/j.1523-1755.2004.00933.x

[16] Marshall MR Vandal AC de Zoysa JR , et al. Effect of low-sodium versus conventional sodium dialysate on left ventricular mass in home and self-care satellite facility hemodialysis patients: a randomized clinical trial. J Am Soc Nephrol 2020; 31(5): 1078–1091. 32188697 10.1681/ASN.2019090877 PMC7217404

[17] Mendoza JM Bayes LY Sun S , et al. Effect of lowering dialysate sodium concentration on interdialytic weight gain and blood pressure in patients undergoing thrice-weekly in-center nocturnal hemodialysis: a quality improvement study. Am J Kidney Dis 2011; 58(6): 956–963. 21875769 10.1053/j.ajkd.2011.06.030 PMC4124938

[18] Ledebo I. On-line preparation of solutions for dialysis: practical aspects versus safety and regulations. J Am Soc Nephrol 2002; 13: 78–83. 11792766

[19] Flanigan MJ. Role of sodium in hemodialysis. Kidney Int 2000; 58(76): 72–78. 10.1046/j.1523-1755.2000.07609.x 10936802

[20] Ságová M Wojke R Maierhofer A , et al. Automated individualization of dialysate sodium concentration reduces intradialytic plasma sodium changes in hemodialysis. Artif Organs 2019; 43(10): 1002–1013. 30939213 10.1111/aor.13463 PMC6850400

[21] Canaud B Kooman J Selby NM , et al. Sodium and water handling during hemodialysis: new pathophysiologic insights and management approaches for improving outcomes in end-stage kidney disease. Kidney Int 2019; 95(2): 296–309. 30665570 10.1016/j.kint.2018.09.024

